# Oral Anticoagulants Initiation in Patients with Atrial Fibrillation: Real-World Data from a Population-Based Cohort

**DOI:** 10.3389/fphar.2017.00063

**Published:** 2017-02-17

**Authors:** Clara L. Rodríguez-Bernal, Isabel Hurtado, Aníbal García-Sempere, Salvador Peiró, Gabriel Sanfélix-Gimeno

**Affiliations:** ^1^Health Services Research Unit, Fundación para el Fomento de la Investigación Sanitaria y Biomédica de la Comunidad ValencianaValencia, Spain; ^2^Red de Investigación en Servicios de Salud en Enfermedades CrónicasValencia, Spain

**Keywords:** anticoagulants, atrial fibrillation, stroke prevention, pharmacoepidemiology, drug prescription, real-world data, non-VKA oral anticoagulants

## Abstract

**Objective:** Little is known about initial prescription of currently used oral anticoagulants (OAC), and correlated characteristics in real-world practice. We aimed to assess patterns of initiation of Vitamin K antagonists (VKA) and non-VKA oral anticoagulants (NOAC) in naive patients with non-valvular atrial fibrillation and the factors associated with starting treatment with NOAC.

**Methods:** Population-based retrospective cohort study of all patients with NVAF who had a first prescription of OAC from November 2011 to February 2014 in the Valencia region, Spain (*n* = 21,881). Temporal trends of OAC initiation are described for the whole population and by type of OAC and therapeutic agent. Factors associated with starting treatment with NOAC (vs. VKA) were identified using logistic multivariate regression models.

**Results:** Among the patients initiating OAC, 25% started with NOAC 2 years after market release. Regarding temporal trends, prescription of NOAC doubled during the study period. VKA prescription also increased (by around 13%), resulting in a 30% rise in total treatment initiation with OAC during 2011–2014. NOAC initiation (vs. VKA) was associated with a lower baseline risk of thromboembolism and higher income.

**Conclusions:** In this Spanish population-based cohort, initiation of OAC therapy saw a rapid increase, mainly but not exclusively, due to a two-fold rise in the use of NOAC. Initiation with NOAC was associated with a lower baseline risk of thromboembolism and higher income, which opposes the indications of NOAC use and reflects disparities in care. Inadequate prescription patterns might threaten the effectiveness and safety of these therapies, thus monitoring OAC prescription is necessary and should be setting-specific.

## Introduction

Drug prescription to naïve patients—initial prescription—is the starting point for establishing treatment adequately, and provides an important opportunity to engage patients in therapy. Oral anticoagulation (OAC) significantly reduces the risk of stroke in patients with atrial fibrillation when used adequately (1990; 1991; 1994; Petersen et al., 1989; Lancaster et al., 1991). Vitamin K antagonists (VKA) (Fuster et al., 2012; January et al., 2014), and more recently the non-VKA oral anticoagulants (NOAC), which are comparable in efficacy and safety to VKA (Connolly et al., 2009; Granger et al., 2011; Patel et al., 2011), are the therapy indicated for this purpose. NOAC have some theoretical advantages over VKA such as the scarcity of interactions, predictable effects with fixed dosages and no need for monitoring. Nevertheless, they also have significant limitations, including the unavailability of tests for monitoring their anticoagulant effectiveness, the low availability of antidotes to reverse their effect, and renal clearance. Additionally, their cost far outweighs that of traditional anticoagulation.

Consequently, diverse guidelines recommend the use of NOAC or VKA to reduce the risk of stroke, although different approaches are being used. For example, US guidelines recommend the use of either NOAC or VKA for the prevention of stroke (Wann et al., 2011). NOAC as first line therapy is recommended by European guidelines (Camm et al., 2010, 2012) whereas in Spain, the prescription of NOAC in patients with AF requires prior authorization (AEMPS, 2016). However, very little is known about anticoagulant therapy use or initiation globally. Two recent studies, one set in China—registry-based- and the other in Denmark—population-based-, explored oral anticoagulant use, but not initiation in naïve patients (Adelborg et al., 2016; Chang et al., 2016).

Evidence on initial prescription patterns and their predictors for direct and traditional oral anticoagulants in patients with non-valvular atrial fibrillation, comes from one study in the United States (Desai et al., 2014) and another in Denmark (Olesen et al., 2015), only the latter being population-based.

The current—and ongoing—assessment of initiation patterns and factors related to them might provide useful insights into the appropriateness of initial prescription according to approved indications, as well as data that can be used for posterior surveillance or to study adequately comparative effectiveness in real world practice.

To address this global knowledge gap, we aimed to describe the patterns of prescription of oral anticoagulants (VKA and NOAC) as a starting treatment in naïve patients with non-valvular atrial fibrillation, and to study factors associated with treatment initiation with NOAC in a large Spanish population-based cohort.

## Methods

### Study design

Population-based retrospective cohort study of all patients with atrial fibrillation, who were prescribed oral anticoagulant therapy from November 2011 (date of marketing of first NOAC in Spain) to February 2014 in the Valencia region.

### Population and setting

**Figure 1:**
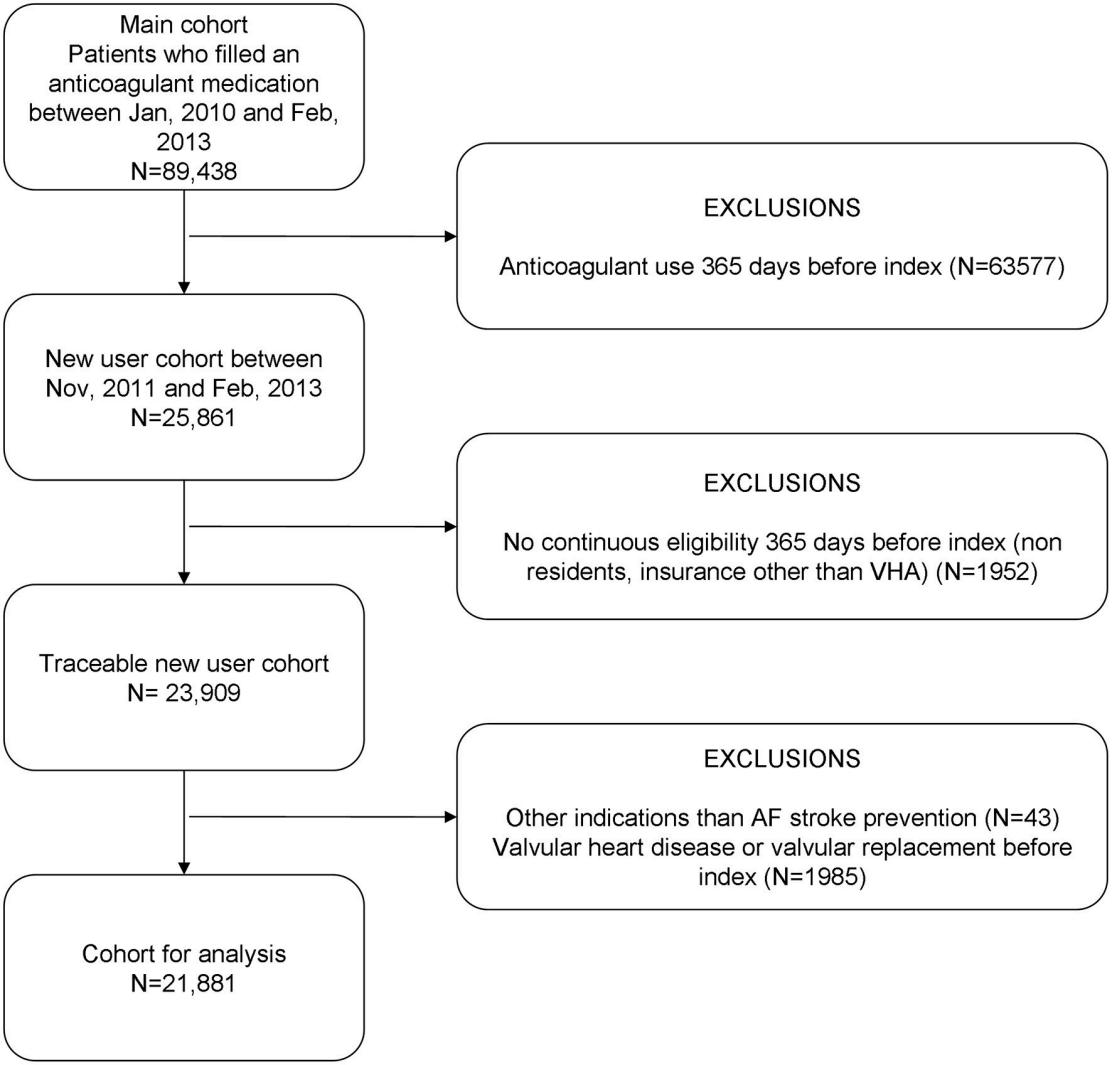
**Eligibility and exclusion criteria to define the study cohort**. Patients served by the Valencia Health System. Spain.

The study was set in the Valencia region and, specifically, in the population covered by the Valencia Health System (VHS), the public health system covering about 97% of the region's population (≈5 million inhabitants). All patients with AF (diagnosis code of International Classification of Diseases, 9th Revision, Clinical Modification [ICD-9-CM] 427.31) newly prescribed with oral anticoagulants (warfarin, acenocoumarol, dabigatran, rivaroxaban, apixaban) for the prevention of thromboembolic events between November 2011 and February 2014 were included. We defined as naïve those patients without anticoagulant treatment in the 12 months preceding the index date (first prescription). People without pharmaceutical/health coverage by VHS, mainly certain government employees whose prescriptions are reimbursed by civil service insurance mutualities, and thus not included in the pharmacy databases of the VHS, and patients not registered in the municipal census (non-residents or temporary residents), were excluded because of limitations on follow-up. We also excluded patients with concomitant valvular heart disease (ICD-9: 394.x-397.x, 398.9, 42.4x, V42.2, V43.3, 35.1x, 35.2x). This left a total of 21,881 patients included in the study (Figure 1).

### Data sources

The main source of data was the VHS ambulatory electronic medical record (EMR), the so-called ABUCASIS system, which among other information includes demographic and clinical data, and information on prescriptions and dispensations. The information on hospitalizations was based on the Minimum Basic Dataset (MBDS) at hospital discharge, a synopsis of clinical and administrative information on all hospital discharges, including diagnoses and procedures. VHS coverage and some demographic characteristics were obtained from the Population Information System. A detailed description of the data sources is found elsewhere (Sanfélix-Gimeno et al., 2015).

### Covariates

We included patients' sociodemographics and a wide range of data on patients' comorbidities, drug, and health care utilization. Sociodemographic data included age, sex, country of origin, and income. Information on baseline comorbidities was obtained by taking into account the 365 days before the index date. The following covariates were considered: Congestive heart failure, hypertension, diabetes, liver, and renal disease, previous ischemic stroke or TIA, coronary artery disease, deep vein thromboembolism or pulmonary embolism, hemorrhagic stroke, gastrointestinal bleeding, other major bleeding and bleeding history, or predisposition. The risk of major bleeding was measured with the HAS-BLED score, and CHADS2 or CHA2DS2-VASc (Gage et al., 2001; Lip et al., 2010; Pisters et al., 2010) were used to assess the baseline risk of thromboembolic events. Regarding drug-related variables, electronic dispensing (e-prescriptions transmitted directly to the pharmacy) was included. Indicators of health care utilization in the previous year were also estimated: the number of medications, hospitalizations, emergency department visits, visits to outpatient care (cardiologist, neurologist), social worker, nurse and mental health. Hospitalization 30 days before treatment initiation was also included.

### Ethics statement

The study protocol was approved by the Clinical Research Ethics Committee of the “Dirección General de Salud Pública” y “Centro Superior de Investigación en Salud Pública” and reviewed and classified by the Spanish Agency of Pharmacy and Medical Products. Data was sent by the VHS to researchers with dissociate non-traceable codes that would not allow the identification of individual patients. The exemption of informed consent was approved by the Ethics Committee considering the characteristics of the study.

### Statistical analysis

Cohort characteristics were described according to the type of OAC prescribed as starting treatment (VKA vs. NOAC). Temporal trends of OAC initiation (patients starting OAC treatment per month during the study period) were plotted for the whole population, and according to the therapeutic agent, as well as to the type of OAC (VKA/NOAC). Logistic multivariable regression models were constructed to identify predictors of anticoagulation initiation with NOAC (vs. VKA). Backward forward stepwise methods were used to remove nonsignificant variables (with a removal probability of 0.10 and an entry probability of 0.05). The goodness-of-fit was evaluated using the C-Statistic (the area below the receiver operating characteristic [ROC] curve) for discrimination and the Hoshmer-Lemeshow test for calibration. Additionally, we calculated the mean value of the CHADS score by therapeutic group and for total OAC prescription along the study period. All statistical analyses were performed using the Stata 13 (Stata Corp, College Station, TX, USA) statistical software.

## Results

### Cohort characteristics

Our population comprised 21,881 patients, of whom 18% were prescribed NOAC for the initiation of anticoagulation therapy, overall (Table 1). Mean age was 74.5 years and 48% of the population were females. Around 14% of patients had a previous stroke, and the cohort had a mean score of 2.2 for both the CHADS2 and HAS-BLED scores.

**Table 1:** **Characteristics of patients with non-valvular atrial fibrillation starting OAC therapy, and by VKA and NOAC**.

	**OAC**	**VKA**	**NOAC**
*N* (%)	21.881	17.948 (82.03)	3.933 (17.97)
Female (%)	47.65	47.7	47.42
Age, years, mean (*SD*)	74.50 (10.08)	74.56 (9.86)	74.22 (11.06)
**COUNTRY (%)**			
Spain	96.49	96.54	96.26
European	3.29	3.23	3.56
Non-European	0.22	0.23	0.18
**ANNUAL INCOME, €, (%)**			
<18,000	83.72	84.79	78.88
18,000–100,000	15.99	14.58	20.51
>100,000	0.29	0.22	0.61
**COMORBIDITIES (%)**			
Congestive heart failure	21.33	22.00	18.28
Hypertension	79.35	79.70	77.73
Diabetes	30.91	31.68	27.41
Liver disease	6.22	6.36	5.57
Renal disease	11.77	12.55	8.21
Previous ischemic stroke or TIA	14.47	13.96	16.83
Coronary artery disease	20.96	21.21	19.86
Deep vein thromboembolism or pulmonary embolism	6.09	6.48	4.30
Hemorrhagic stroke	0.82	0.70	1.37
Gastrointestinal bleeding	3.66	3.69	3.51
Other major bleeding	20.60	20.84	19.48
Bleeding history or predisposition	23.19	23.35	22.48
CHADS2 score, mean (*SD*)	2.18 (1.25)	2.19 (1.26)	2.12 (1.34)
CHA2DS2-VASC score, mean (*SD*)	3.85 (1.70)	3.86 (1.70)	3.71 (1.82)
HAS-BLED score, mean (*SD*)	2.23 (1.00)	2.23 (1.00)	2.16 (1.03)
**HEALTHCARE UTILIZATION, MEAN (*SD*)**			
Number of medications	9.89 (4.82)	9.96 (4.86)	9.57 (4.68)
Hospitalizations	0.66 (0.94)	0.68 (0.96)	0.61 (0.90)
Emergency department visits	1.22 (1.49)	1.25 (1.50)	1.07 (1.46)
Outpatient visits			
Cardiologist visits	0.47 (0.85)	0.43 (0.82)	0.66 (0.98)
Neurologist visits	0.14 (0.53)	0.14 (0.51)	0.19 (0.65)
Family physician visits	10.36 (7.28)	10.37 (7.20)	10.32 (7.68)
Social work visits	0.09 (0.68)	0.09 (0.68)	0.09 (0.70)
Mental health visits	0.09 (0.84)	0.09 (0.81)	0.11 (0.96)
Nurse visits	8.54 (11.57)	8.74 (12.01)	7.65 (10.59)
Hospitalization in 30 days before treatment initiation (%)	30.82	31.41	28.12

*Retrospective cohort of naïve patients in the Valencia Region. Data presented as percentages or means (SD). OAC indicates oral anticoagulants (both VKA and NOAC); VKA, Vitamin K antagonists NOAC, Non-VKA oral anticoagulants; TIA, transient ischemic attack*.

VKA initiators were more likely to have higher risk scores, more comorbidities and higher health services utilization, overall. NOAC initiators were more likely to have higher income, previous ischemic stroke and hemorrhagic stroke, and more visits to cardiologist and neurologist.

### Temporal trends

**Figure 2:**
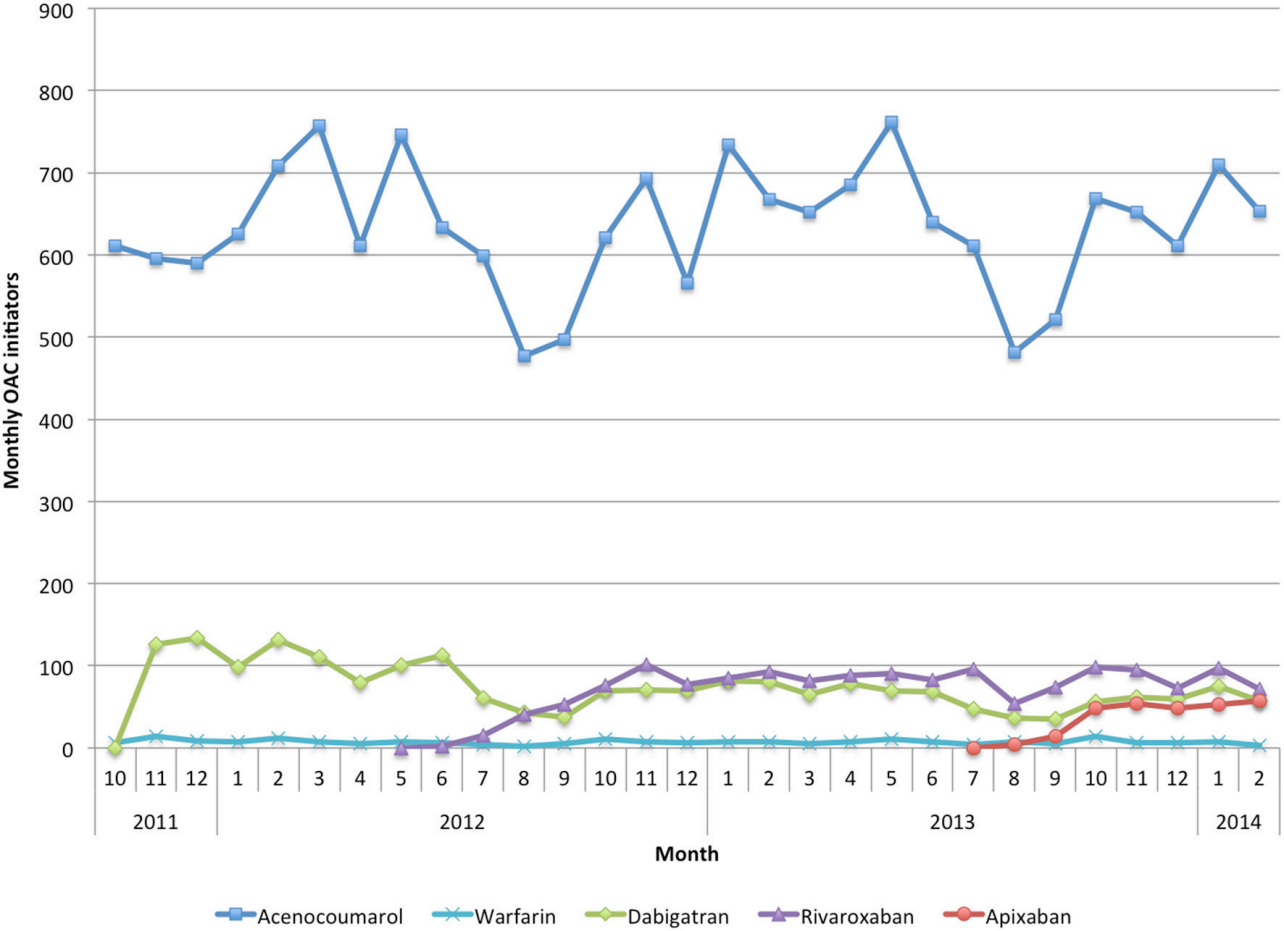
**The *x* axis represents the months during the study period**. The *y* axis represents the number of patients initiating OAC therapy.

Prescription rates of acenocoumarol as starting treatment did not seem to be affected by the introduction of NOAC (Figure 2). Concurrently, each NOAC showed a steep rise after its introduction onto the market, leveling out afterwards. The introduction of each new NOAC seemed to slightly affect the market share of those already available. However, they experienced a recovery to their initial levels, with no apparent substitution effect among them. Apixaban seemed to experience a more modest increase than the previous two NOACs (rivaroxaban and dabigatran), although apixaban was available for a shorter period in our study.

**Figure 3:**
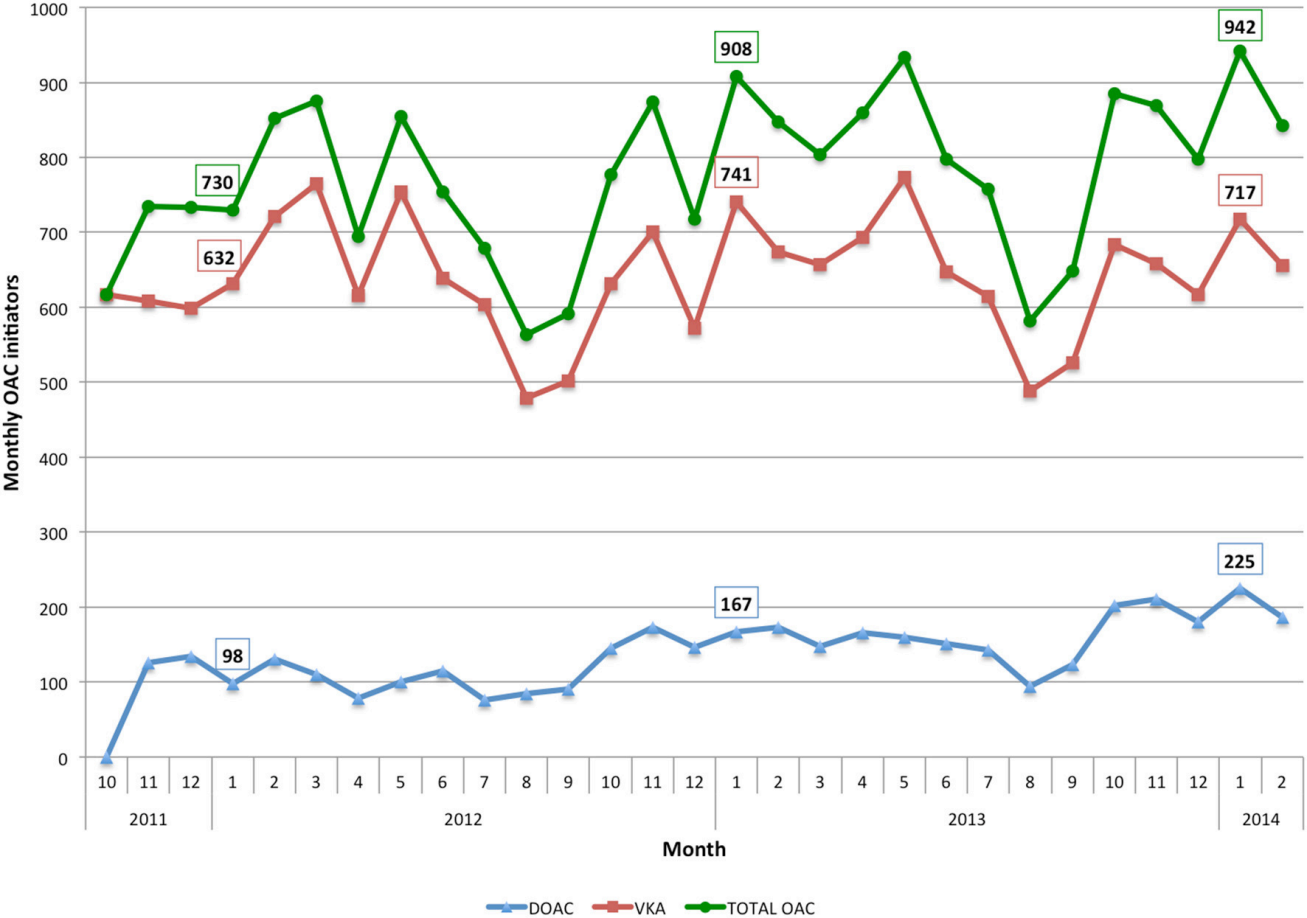
**The *x* axis represents the months during the study period**. The *y* axis represents the number of patients initiating OAC therapy.

Temporal trends in total OAC initiation, and by therapeutic class are shown in Figure 3. The number of patients starting anticoagulation treatment with NOAC more than doubled during the period of study (from 97 new patients per month in January 2012 to 225 in January 2014). In relative terms, 2 years after the introduction onto the market of the first NOAC (i.e., November 2013, as NOAC were released in November 2011), 25% of patients with AF starting OAC therapy were being prescribed NOAC. The number of patients prescribed VKA as initial treatment also increased, by around 13% (from 632 new patients per month in January 2012 to 717 in January 2014). Overall, a cumulative rather than a substitutive effect was observed, resulting in a rise of around 30% in the total OAC initiation rate during the study period (730 new patients started treatment in January 2012 as compared to the 942 who did so in January 2014). Furthermore, from the total number of patients starting OAC therapy, the percentage starting with NOAC rose from 13.4% in January 2012 to 23.9% in January 2014.

### Predictors of OAC initiation

In the adjusted analysis, the initiation of therapy with NOAC was positively associated with annual income, older age (66–75 vs. ≤ 65 years), previous stroke, intracranial hemorrhage, and at least one visit to the cardiologist and neurologist; patients with renal and coronary artery disease, diabetes, deep vein thromboembolism, and at least two emergency department visits were less likely to initiate with NOAC (Table 2).

**Table 2:** **Factors associated with NOAC initiation (vs. VKA)**.

	**OR**	**95% CI**	***p*****-value**
Age, 65–74 y (ref. < 65)	0.87	0.81–0.94	0.001
**ANNUAL INCOME, €, (%)**			
<18,000	1	1	
18,000–100,000	1.41	1.28–1.54	<0.0001
>100,000	2.63	1.55–4.46	<0.0001
**COMORBIDITIES (%)**			
Congestive heart failure	0.91	0.83–0.99	0.039
Diabetes	0.85	0.79–0.92	<0.0001
Renal disease	0.68	0.60–0.77	<0.0001
Previous ischemic stroke or TIA	1.31	1.18–1.45	<0.0001
Coronary artery disease	0.90	0.82–0.99	0.024
Deep vein thromboembolism or pulmonary embolism	0.71	0.60–0.83	<0.0001
Hemorrhagic stroke	1.93	1.39–2.69	<0.0001
**HEALTHCARE UTILIZATION**			
Emergency department visits (ref. <2)	0.78	0.73–0.84	<0.0001
Ambulatory visits			
Cardiologist visits	1.19	1.05–1.34	<0.0001
Neurologist visits	1.86	1.73–2.00	0.005

*Retrospective cohort of naïve patients in the Valencia Region. NOAC indicates non-VKA oral anticoagulants; VKA, Vitamin K antagonists; OR, Odds Ratio; TIA, transient ischemic attack. Stepwise multivariable logistic regression. Only statistically significant covariates retained in the model are presented. All covariates (as presented in Table 1) were included in the model. Pseudo R2 = 0.03; p < 0.0001; C-Statistic: 0.62; p(x2) Hosmer-Lemeshow = 0.3739*.

## Discussion

In the present study, 25% of patients with AF starting OAC therapy were being prescribed NOAC as initial treatment 2 years after the introduction onto the market of the first NOAC. Overall, we found that initiation with NOAC doubled during the study period. VKA initiation also increased (by around 13%) with no apparent substitution effect by NOAC. Therefore, total treatment initiation with OAC rose by a staggering 30% during the study period. Regarding temporal trends in the initiation of NOAC, steep rises were observed for dabigatran and rivaroxaban after their introduction, followed by stabilization, whereas apixaban (the last drug entering the market) seems to have had a more modest increase (although it had a shorter observation period). The most important predictors of NOAC initiation (vs. VKA) were a lower baseline risk of thromboembolic events (based on stroke risk factors) and higher income.

To our knowledge, only two studies have assessed OAC (VKA and NOAC) initiation patterns in real-world practice to date (Desai et al., 2014; Olesen et al., 2015). The most remarkable finding in our population-based cohort is that, apart from the important increase in the prescription of NOAC as starting treatment, VKA prescription also increased, resulting in an important rise in total treatment initiation with OAC, with no apparent substitution effect; while the US (Desai et al., 2014) and the Danish (Olesen et al., 2015) studies showed a decline in rates of VKA use and a considerable uptake in NOAC, suggesting a shift from VKA to NOAC. Approximately 2 years after the introduction onto the market of the first NOAC, in the US and Danish studies around 60% of patients initiating oral anticoagulation therapy did so with NOAC, while in our study around 24% of patients started therapy with NOAC.

These discrepant findings are likely to be due to the differences among practice guidelines and barriers for NOAC prescription in Spain. While the European Society of Cardiology has expressed a preference for NOAC over VKA in stroke prevention for AF patients (Camm et al., 2010, 2012) and the ACC/AHA/HRC guidelines have the same level of recommendation for VKA and NOAC (Wann et al., 2011; January et al., 2014), in Spain NOAC are recommended as a second line treatment and their prescription requires prior authorization.

Regarding the increase in total OAC prescription as starting treatment, a plausible explanation is that in our population, the threshold for OAC initiation may have decreased over time. However, when assessing CHADS_2_ scores yearly and by drug, we observed that they increased over time in both, NOAC and VKA users (Supplementary Figure 1), suggesting that either more high-risk patients have been treated, or that due to the prior authorization necessary in the case of NOAC prescription, physicians are prone to register comorbidities more accurately, overall. Another possible explanation is that OAC therapy has been recently in the focus of scientific attention and marketing strategies, resulting in a global increase in OAC prescription. Regardless of the explanation, this, overall, probably implies a potential improvement in care in our setting, given that in our study the risk profile of patients treated—as measured by the CHADS2 score-increased over time (Supplementary Figure 1), suggesting that high-risk patients were being under-treated.

Regarding factors related to treatment initiation with NOAC (vs. VKA), lower rates of renal disease and higher rates of previous hemorrhagic stroke were found in NOAC patients, as expected. Although not directly comparable, the findings of two previous studies are similar (Brais et al., 2015; Patel et al., 2015). Coronary artery disease in our study was inversely associated with NOAC initiation, probably due to controversial evidence about higher myocardial infarction risk with dabigatran therapy (Douxfils et al., 2014; Giglio et al., 2014). However, Patel et al. found no association between NOAC prescription and coronary disease (Patel et al., 2015).

Furthermore, prescription of NOAC as starting treatment was associated overall with a lower baseline risk of thromboembolic events (as indicated by stroke risk factors) and higher income. These findings are supported by those of Desai et al. (2014) and suggest an indication bias that opposes the indication of NOAC prescription among patients with a higher risk of stroke or other thromboembolic events, which according to the evidence will benefit the most of the use of NOAC as compared with VKA.

This situation highlights the need to monitor the use and appropriateness of OAC prescriptions and to study the comparative effectiveness and safety, costs and cost-effectiveness of OAC agents in real world clinical practice. Such assessments will add important insights for decision-making among stakeholders, medical professionals, and patients.

### Study strengths and limitations

Our study has reliable data on clinical characteristics including diagnoses and procedures, health services utilization, as well as prescription and dispensing; and individual-level data on sociodemographics, retrieved through the linkage of several electronic databases including EMRs, unlike previous studies which data is based on administrative claims. Besides, our cohort is population based. It uses data of the population covered by the public health system, which virtually covers the totality of the inhabitants of the region. These are important advantages over previous studies assessing initiation of oral anticoagulation therapy in real world practice.

The most important limitation of our study is that information biases due to absent registration or differing data recording practices in the EMR might exist, although this is an inherent problem of any study using data from routine clinical practice. Moreover, misclassification (on exposure and covariates) is expected to be non-differential across groups of study subjects. Additionally, when constructing the CHADS risk score, INR was not included, given the unavailability of data for INR values. However, it is unlikely that scores have been affected to the extent of misclassifying patients along the score ranges. Although relevant predictors of NOAC initiation have been identified, the discriminatory capacity of the regression model is low, suggesting that other non-identified factors are driving the selection of NOAC (vs. VKA) as starting treatment.

## Conclusions

NOAC prescription as initial treatment in patients with non-valvular atrial fibrillation rose considerably, doubling during the study period. Furthermore, VKA prescription also increased, leading to a staggering 30% increase in total OAC prescription. NOAC initiation was associated with a lower baseline risk of stroke and higher income. This opposes the indications of NOAC use and reflects disparities in care, indicating the need for a close monitoring of the appropriateness of OAC prescription.

## Author contributions

GS conceived the study and carried out the main statistical analyses. CR drafted the manuscript with relevant input from GS. IH prepared the database and assisted with statistical analysis. CR, IH, AG, SP, and GS participated in the study design and interpretation of data, and contributed to the critical revision of the manuscript for important intellectual content. All authors agree to be accountable for all aspects of the work and have read and approved the final manuscript.

## Funding

This study was partially funded by the 2013 Collaboration Agreement between the *Fundación para el Fomento de la Investigación Sanitaria y Biomédica* (FISABIO) from the Valencia Ministry of Health and Boehringer Ingelheim, a non-conditioned program to conduct independent research in chronic healthcare, pharmacoepidemiology, and medical practice variation. It was also partially funded by the FISABIO grant for Emerging Researchers UGP-15-226. CLRB was funded by the grant RD12/0001/0005 from the *Instituto de Salud Carlos III*, Spanish Ministry of Health (cofinanced by the European Regional Development Fund). The views presented here are those of the authors and not necessarily those of the FISABIO Foundation, the Valencia Ministry of Health or the study sponsors.

### Conflict of interest statement

SP has received fees for participation in scientific meetings and courses sponsored by Novartis and Ferrer International. GS participated in 2014 in an advisory meeting of Boehringer Ingelheim. AG is a former employee of Boehringer Ingelheim. CR and IH have reported that they have no relationships (as individuals) relevant to the contents of this paper to disclose. The collaboration agreement between Boehringer Ingelheim and the authors' institution is reported in all of the disclosures forms. None of the sponsors were involved in the design and conduct of the study; the collection, management, analysis, and interpretation of the data; or the preparation, review or approval of the manuscript, or in the decision to submit it for publication. The other authors declare that the research was conducted in the absence of any commercial or financial relationships that could be construed as a potential conflict of interest.

## Supplementary material

Supplementary Figure 1 **Mean CHADS2 score according to type of OAC**.
