# Supplementary figures and images for: Oral Anticoagulants Initiation in Patients with Atrial Fibrillation: Real-World Data from a Population-Based Cohort
Source: Front Pharmacol. 2017 Feb 17;8:63. doi: 10.3389/fphar.2017.00063 (PMC5314137; doi:10.3389/fphar.2017.00063)

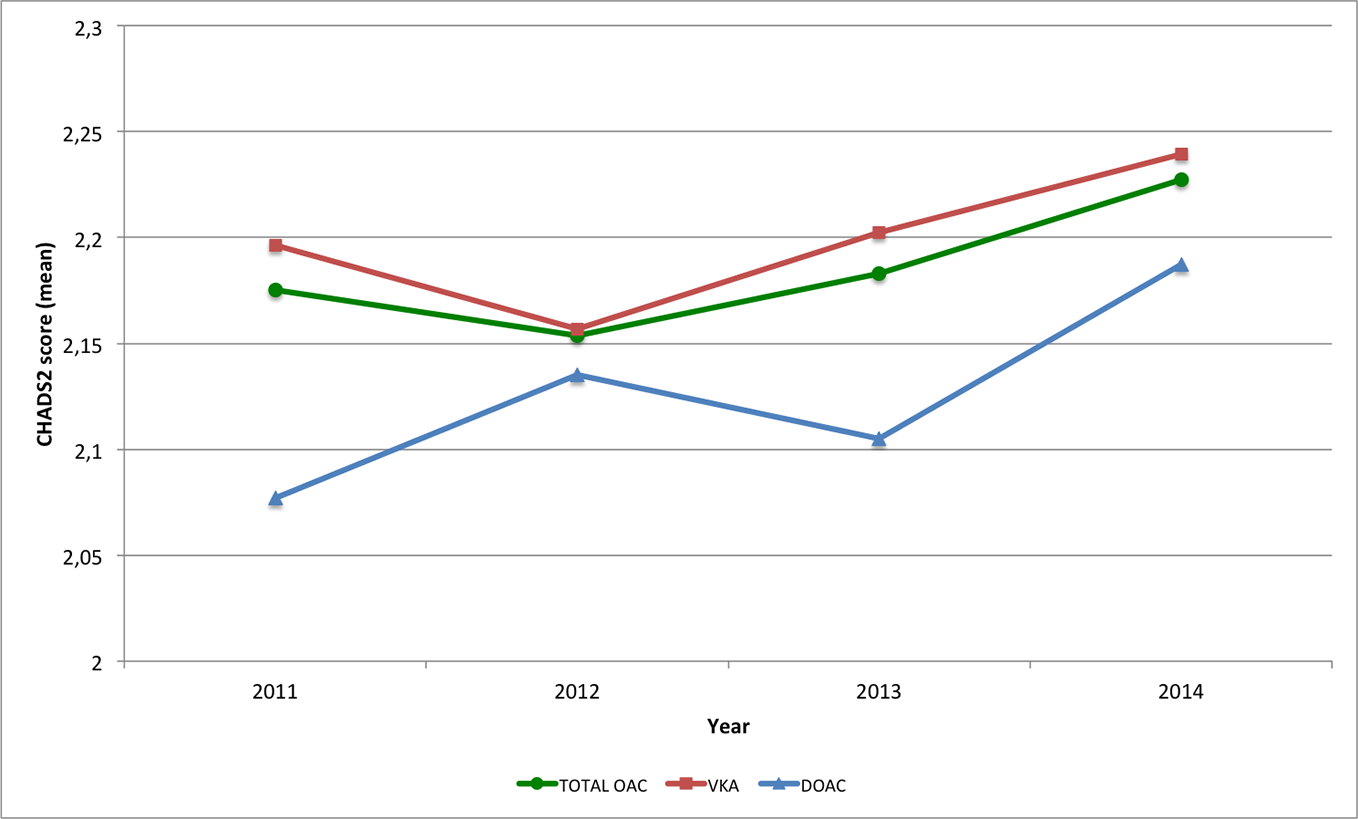

Supplement: Supplementary Figure 1 — Mean CHADS2 score according to type of OAC. [file Image1.TIF]
